# Analysis of exosomal circRNAs upon irradiation in pancreatic cancer cell repopulation

**DOI:** 10.1186/s12920-020-00756-3

**Published:** 2020-07-29

**Authors:** Yi-yun Chen, Ming-jie Jiang, Ling Tian

**Affiliations:** 1 Shanghai Key Laboratory of Pancreatic Diseases & Institute of Translational Medicine, Shanghai General Hospital, Shanghai Jiao Tong University School of Medicine, Science bldg. Rm 205, New Songjiang Rd No.650, Songjiang District, Shanghai, 201620 China; 2 College of Chemistry, Chemical Engineering and Biotechnology, Donghua University, Shanghai, 201620 China; 3 Department of Central Laboratory, Shanghai Chest Hospital, Shanghai Jiao Tong University School of Medicine, Shanghai, 200030 China

**Keywords:** Exosome, Circular RNA, Cancer repopulation, Pancreatic cancer, Irradiation, Gene expression profiling

## Abstract

**Background:**

Pancreatic cancer is one of the most malignant tumors. However, radiotherapy can lead to tumor recurrence, which is caused by the residual surviving cells repopulation stimulated by some molecular released from dying cells. Exosomes may mediate cell-cell communication and transfer kinds of signals from the dying cells to the surviving cells for stimulating tumor repopulation. Circular RNAs (circRNAs) may be one vital kind of exosomal cargos involving in modulating cancer cell repopulation.

**Methods:**

Next generation sequencing (NGS) and bioinformatics were performed to analyze and annotate the expression and function of exosome-derived circRNAs in pancreatic cancer cells after radiation. Four circRNAs were chosen for qRT-PCR analysis to validate the sequencing results.

**Results:**

In this study, 3580 circRNAs were annotated in literatures and circBase among 12,572 identified circRNAs. There were 196 filtered differentially expressed circRNAs (the up-regulation and down-regulation respectively is 182 and 14, fold change > 2, *p*-value < 0.05). Regulation of metabolic process and lysine degradation were the main enriched biological processes and pathway according to Gene Ontology (GO) and Kyoto Encyclopedia of Genes and Genomes (KEGG) analysis.

**Conclusions:**

The hsa_circ_0002130-hsa_miR_4482-3p-NBN interaction network suggested potential sponging miRNA and target mRNA. Our results provided potential functions of circRNAs to explore molecular mechanisms and therapeutic targets in pancreatic cancer cell repopulation upon irradiation.

## Background

Pancreatic cancer is a highly fatal disease, which morbidity is almost the same as the mortality. Focusing on the global burden of cancer worldwide, the number of new cases and deaths of pancreatic cancer respectively was 458,918 and 432,242, taking up 2.5 and 4.5% of all the analyzed cancers in 185 countries [1]. The high mortality of pancreatic cancer might partly ascribe to the lack of effective strategies. Radiotherapy should be an optimal choice for those regional unresectable disease without detectable metastasis in most cancers. Although American Society for Radiation Oncology (ASTRO) recently issued a guideline for treatment of pancreatic cancer patients using radiotherapy, either conventional radiotherapy or SBRT, etc., the quality of evidence for recommendation using of radiotherapy was largely low [2]. Efforts to improve the efficacy of radiotherapy in pancreatic cancer is ongoing. New radiation dose, modality, fraction size, and sequencing, as well as in combination with other treatment strategies such as immunotherapy have been largely studied [3]. However, no significant progress has been achieved so far.

As one of the 5Rs of radiobiology, tumor accelerated repopulation after radiation was known as the main cause of treatment failure [4, 5]. Researches by our team have revealed that cancer cell repopulation after radiotherapy were related to the dying cells caused by the treatment, which could inactivate or activate relevant signaling to stimulate the residual surviving cells to fast grow. The related molecules, such as PGE2, microRNAs and other molecules, played as a switch to promote the repopulation [6–8]. Exosomes, one type extracellular vesicle of about 30–150 nm, contain rich cargos such as DNA, mRNA and some non-coding RNAs [9]. Recently, it has been well reported that exosomes might transmit the key factors to the receptor cells, which promote tumorigenesis and progression [10, 11]. Thus, further exploring the contents of pancreatic cancer cell-derived exosomes would help to reveal the molecular mechanism of repopulation upon irradiation.

CircRNAs are predominantly in the cytoplasm, which formed a covalently closed continuous loop without 5’cap or 3’ polyadenylated tail [12]. Therefore, they are resistant towards exonucleases. Based on their biogenesis mechanism, different kinds of circRNAs could be characterized to mainly five parts, exonic circRNAs, intronic circRNAs, antisense circRNAs, sense overlapping circRNAs and intergenic circRNAs [13]. Among them, the exonic circRNAs were the most detected [14]. As endogenous competitive RNAs, circRNAs can compete for miRNAs through MREs (miRNA recognition elements, MREs) [15, 16]. Recently, circRNAs could be sorted into exosomes and participate in cancer progression [17]. Especially, exosome-derived circRNAs could promote invasive growth through miRNA sponge in pancreatic cancer [18]. Although sponging miRNAs has been partly demonstrated during radiation, the function of circRNAs remains largely unknown.

**Fig. 1:**
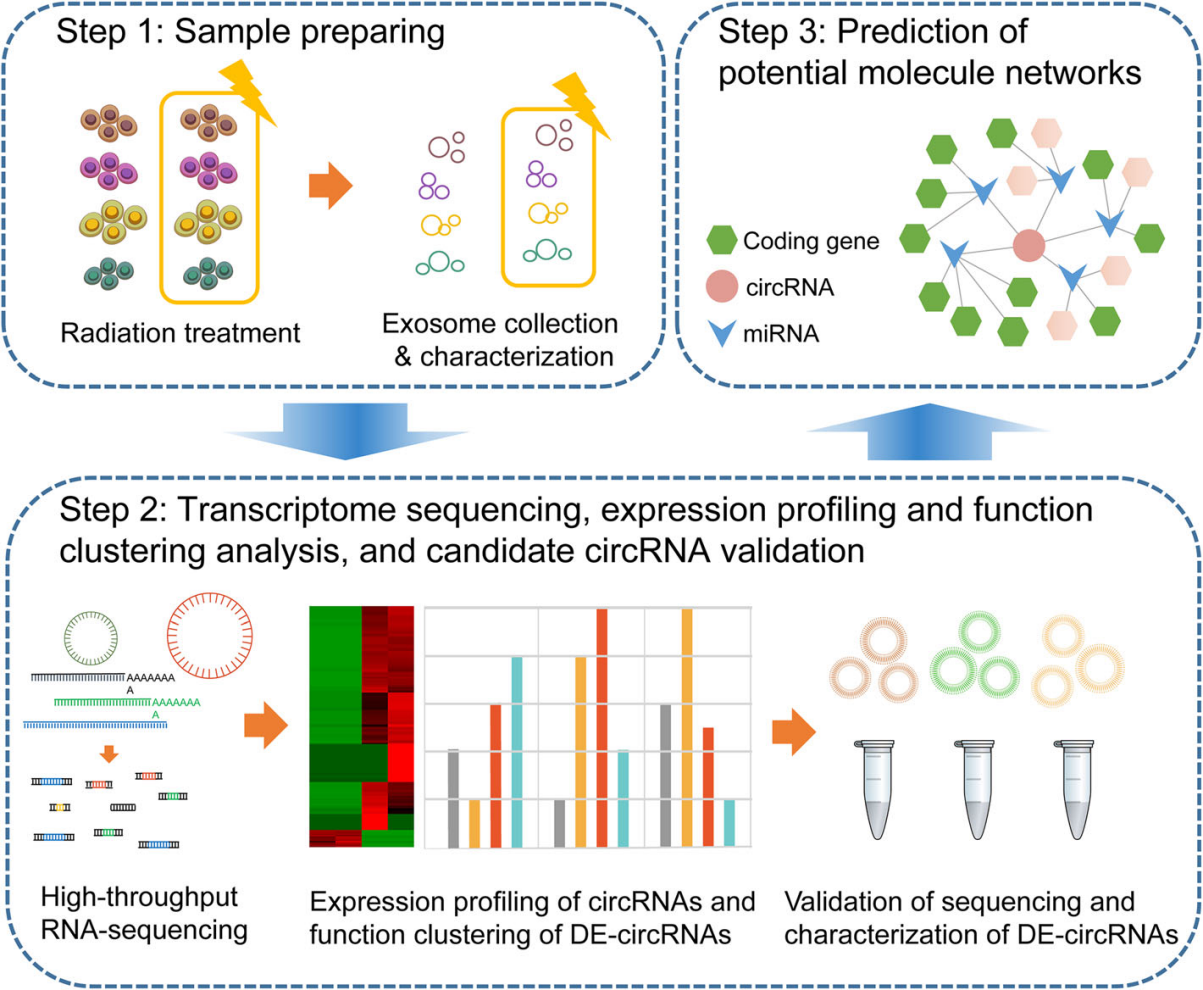
A brief workflow. In the chart, our work was mainly divided into three steps. Through these steps, potential circRNAs were harvested. CircRNA profiling indicated that dying cell derived-exosome carried circRNAs that could regulate modulators and influence the tumor repopulation in pancreatic cancer

During last decades, next generation sequencing (NGS) has been widely used to identify the differentially expressed genes, annotate functional pathways at genomic, transcriptional and epigenetic level, which greatly assists clinicians in early diagnosis and screening of high-risk populations, and eventually allows the development of personalized therapy in pancreatic cancer [19, 20]. In this study, we generated RNA sequencing data from 4 types of pancreatic cancer cells (PANC-1, SW1990, BxPC-3, MIA PaCa-2), which were treated with or without irradiation, and identified ~ 12,570 circRNAs. The accuracy of sequencing was verified by quantitative real-time RT-PCR (qRT-PCR) of the differentially expressed circRNAs (DE-circRNAs). Bioinformatics analysis including GO and KEGG were then performed to annotate the selected DE-circRNA functions. A circRNA-miRNA-mRNA network was subsequently constructed to reveal the molecular regulatory networks. In brief, the whole work flow of our study was shown in Fig. 1.

## Methods

### Cell culture and treatment

Four pancreatic cancer cells were used for analysis, including PANC-1 (ATCC® CRL-1469), BxPC-3 (ATCC® CRL-1687), SW1990 (ATCC® CRL-2172) and MIA PaCa-2 (ATCC® CRL-1420). These cells were divided into two groups, group A as the control group and group B as the experimental group. Ten Gy of X-ray radiation was used in experimental group as we reported to induce tumor repopulation before [6, 7]. Fatal bovine serum (FBS) centrifuged at 120,000 g for 18 h was used for cell culture to avoid the interference with experimental results [21].

### Exosome isolation and identification

Differential centrifugation was used for exosome purification. Briefly, centrifugation was respectively performed at 300, 2000 and 10,000 g to remove the alive cells, dead cells and cell debris. The crude extract of exosomes was obtained by ultra-high speed centrifugation (> 100,000 g), and the operation was repeated twice to remove the contaminated protein for collecting the purified exosomes [22].

### RNA extraction and purification

Total RNAs from two groups (group A and B) were extracted using TRIzol reagent (Life Technologies, USA), and then treated with DNase I (Takara, China) to reduce interference of genomic DNA, according to manufacturer’s instructions. The quantity and purity of total RNAs was determined by NanoDrop ND-100 (Thermo Fisher Scientific, USA). Well qualified RNA was further studied in the following experiments.

### circRNA library construction and sequencing

rRNAs were removed by Ribo-Zero rRNA Removal Kits (Illumina, USA) from total RNAs. TruSeq Stranded Total RNA Library Prep Kit (Illumina, USA) was used for constructing RNA libraries following the manufacturer’s instructions. The quality and quantification of libraries was detected by BioAnalyzer 2100 system (Agilent Technologies, USA). Ten pM libraries were denatured to single-stranded DNA molecules, which amplified in situ as clusters and sequenced for 150 cycles on Illumina HiSeq Sequencer according to the manufacturer’s instructions.

### Sequence mapping and circRNA annotation

Paired-end reads were obtained from Illumina HiSeq 4000 sequencer for quality control by Q30. After 3’ adaptor-trimming, Cutadapt software (v1.9.3) was used to remove low quality reads, and STAR software (v2.5.1b) was used to compare high quality reads with hg19 human reference genome. CircRNAs were detected and identified using DCC software (v0.4.4). EdgeR software (v3.16.5) was used for data normalization and DE-circRNA analysis. GO and KEGG analysis was performed to predict the function of associated genes of candidate circRNAs.

### Identification of DE-circRNAs

DE-circRNAs in the two groups were identified using the edgeR software with quasi-likelihood F test (fold change > 2 and *p*-value < 0.05). The DE-circRNAs were log_2_ transformed for standardization and further visualization. Visual graphics were generated using ggplot2 in R (https://cran.r-project.org/web/packages/ggplot2/index.html).

### Functional annotation of target miRNAs and prediction of interaction networks

In terms of functional annotation of candidate circRNAs, the source genes of DE-circRNAs were clustered in GO annotations (http://www.geneontology.org) and KEGG pathways (http://www.genome.jp/kegg/) by DAVID Bioinformatics Resources (http://david.ncifcrf.gov/home.jsp). *P*-value was calculated by fisher test and adjusted by Benjamini& Hochberg. The result of prediction of potential circRNA-miRNA binding sites was obtained through TargetScan (http://www.targetscan.org) and miRanda (http://www.microrna.org/microrna/home.do). The mRNA-miRNA-circRNA interaction analysis was performed for top 3 expression quantity by using TargetScan, miRanda, Circinteractome (https://circinteractome.nia.nih.gov/) and Circbank (http://www.circbank.cn/). The enrichment score was comprehensively considered to the score from TargetScan and the thermodynamic properties of the binding site from miRanda. The predicted target genes of the DE-circRNAs were further subjected to GO term and KEGG pathway analyses. The gene network analysis was performed using Cytoscape.

### qRT-PCR

Total RNA was reverse transcribed using random primers with the PrimeScript RT reagent kit (RR037A, Takara), following the manufacturer’s protocol. We randomly selected 4 DE-circRNAs, including 3 up-regulated and 1 down-regulated circRNAs. Details of the primer sequences are summarized in Supplementary Table 4. Only primers whose PCR products showed a single peak in the melting curve were considered for qRT-PCR validation. Then, qRT-PCR was performed using SYBR Premix Ex Taq II (Takara, Dalian, China) on a QuantStudio 6 Flex (Life technologies, USA) according to the manufacturer’s instructions. The expression level of candidate circRNAs was normalized to β-actin and then calculated using the 2^-△△Ct^ method. All experiments were repeated three times and presented as the means ±SD.

### RNase R treatment

DNase-treated total RNAs were incubated for 1 h at 37 °C with or without RNase R (Epicentre). We used linear RNA FBXW7, GAPDH, β-actin, 18S rRNA (which are poly(A)-tailed and must be degraded by RNase R treatment) and circRNA FBXW7 as controls.

### Patient-derived xenograft mouse model

All animal experiments were conducted according to protocols approved by Shanghai General Hospital’s Institutional Animal Care and Use Committee (IACUC). Clinical surgical specimens from tumor patients were cut into pieces and subcutaneously implanted into pentobarbital-anaesthetized BALB/c nude mice (about 6-week-old; purchased from Shanghai SLAC Laboratory Animal Co.,Ltd) at right groin to build patient-derived xenograft (PDX) mouse model. When PDX tumor grew into about 500 mm^3^, mice were subjected to 10 Gy radiation or mock treatment (5 mice for each group and sample size was chosen based on routine of cancer investigations). One week after radiation, the mice were anaesthetized with pentobarbital, blood was collected through heart punctures and EDTA was used as the anticoagulation agent. Mice were then euthanized by cervical dislocation. Plasma was separated by centrifuge at 3000 rpm for 15 min. Exosomes were collected from plasma by ultracentrifugation.

### Western blot

Protein was isolated by 10% SDS-polyacrylamide gel electrophoresis (SDS-page) and then transferred to a nitrocellulose membrane (Sigma-Aldrich, USA). After blocking with 5% defatted milk for 90 min, membranes were incubated with TSG101 antibody (1:1000; Abcam, UK) at 4 °C overnight. Membranes were washed and incubated for 1.5 h with secondary antibodies (1:5000; Goat Anti-Rabbit IgG (H + L) Dylight 800, Odyssey, LI-COR, USA). The band intensity was measured using Empiria Studio. GAPDH was used as a loading control.

### Statistical analysis

GraphPad Prism 7.0 was used for statistical calculation (version 7.0c; GraphPad Software, San Diego, USA). All the data were expressed as ± SD of the mean value for three independent measurements. *t* test was used to evaluate the differences between groups. *p*-value < 0.05 were considered statistically significant.

## Results

### Exosome characterization and RNA-seq library construction

**Fig. 2:**
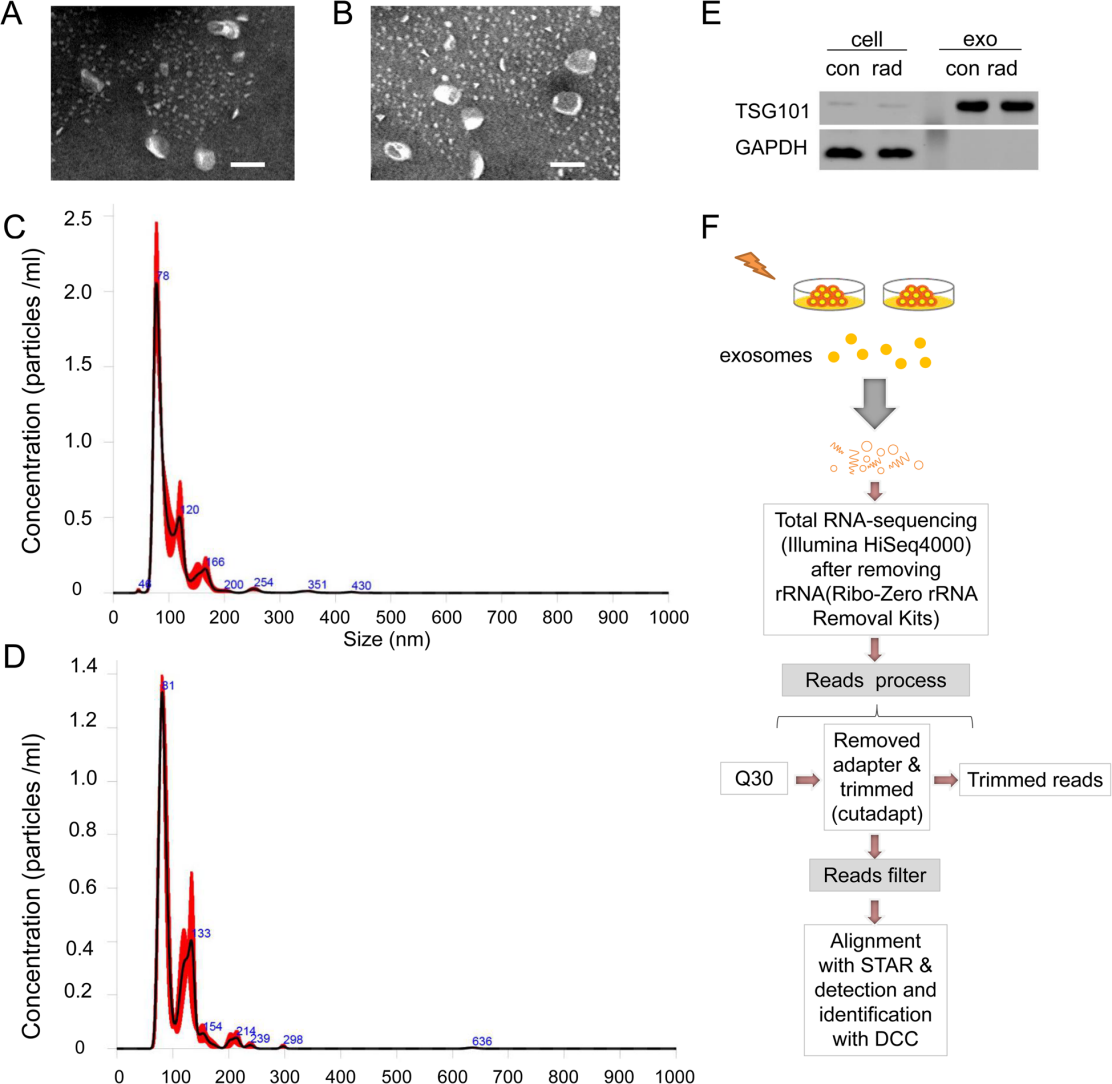
Exosomes identification and RNA-seq libraries construction. a, b Representative TEM image of exosomes in samples. Negative staining was used to enhance the view of membrane structures (bar = 100 nm). **c, d** Representative grain-size graph of exosomes in samples. **e** Representative western blot images of the exosomes and cells. **f** Overview of the comprehensive computational scheme for systematic identification of circRNAs in samples

Exosomes secreted by 4 kinds of pancreatic cancer cells (PANC-1, SW1990, MIA Paca-2, BxPC-3) that were treated with (group B) or without (group A) irradiation were obtained by ultracentrifugation. Under transmission electron microscope (TEM), exosomes were irregular spheres ranging of 30–100 nm in diameter (Fig. 2a, b), which agreed well with the references [9–11]. The concentration of exosomes in irradiation group was higher than those in control one (Fig. 2c, d). Western blot was performed to analyze TSG101, a common biomarker of exosome [22]. Exosomes showed high expression of TSG101 (Fig. 2e), however, there was no expression inside the cells.

To systematically identify circRNAs from exosomes, 4 samples were sequenced using quality-controlled total RNA-seq (rRNA depleted), which generated approximately 380 million reads. A flowchart of sequencing process was shown in Fig. 2f. The quality of RNA was shown in Supplementary Table 1. The quality and quantities of libraries were showed in Supplementary Table 2. More than 86% of 10,096 circRNAs were aligned by UCSC hg19 with STAR software (v2.5.1b) (Supplementary Table 3). Whereafter, the data were normalized and analyzed to identify differentially expressed circRNA.

### Expression profiling of circRNAs

**Fig. 3:**
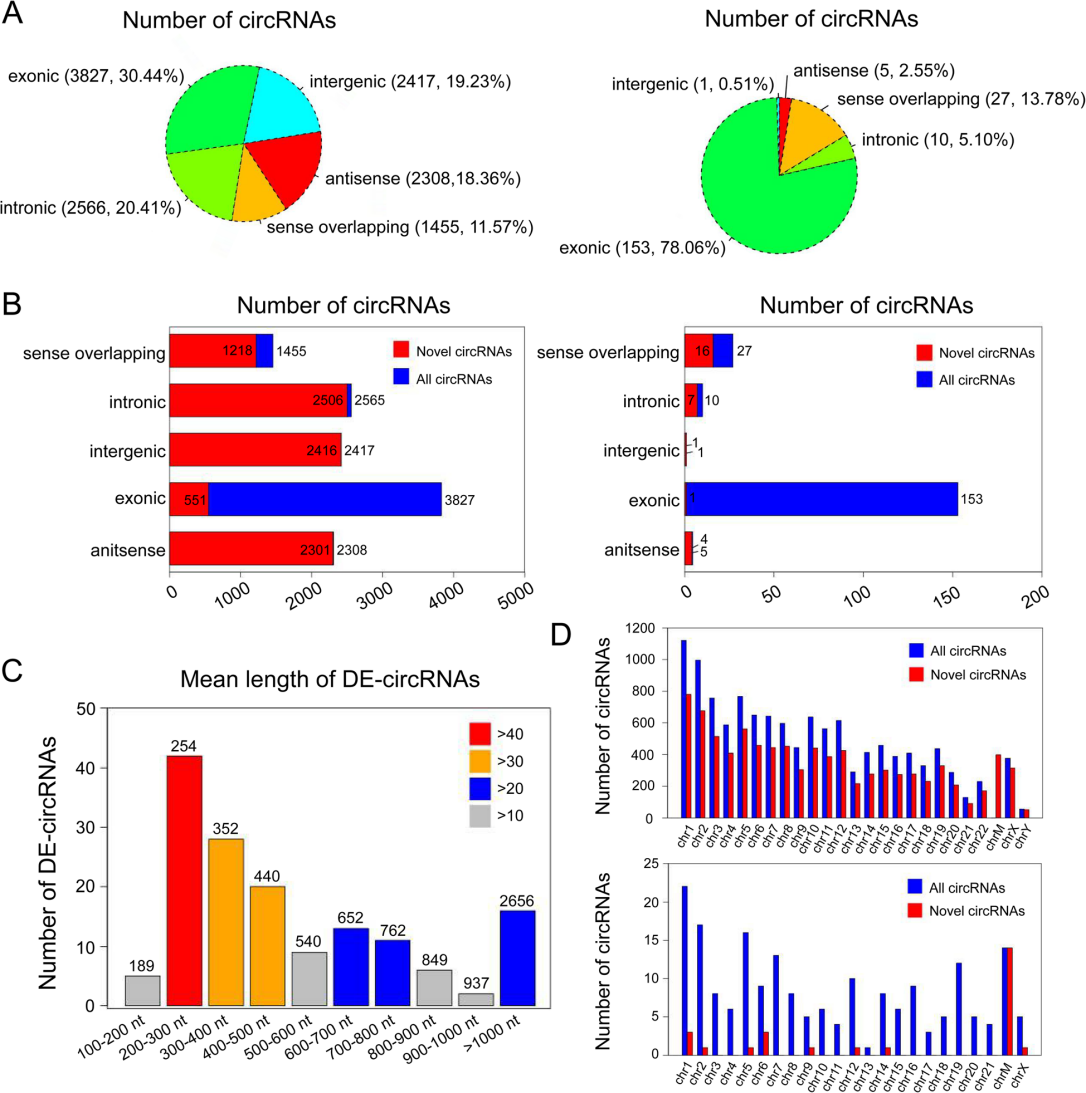
Expression patterns of circular RNAs. a The proportion of 5 isoforms of all predicted and differentially expressed (DE) circRNAs. **b** The further distribution of novel circRNAs among all predicted and DE-circRNAs. **c** The mean length of the exonic circRNAs among the known DE-circRNAs. **d** The chromosomal distribution of novel circRNAs among all predicted and DE-circRNAs

12,572 cicRNAs were quantitated in whole samples, and 8992 of them were novel based on comparison with circBase and published literatures. The majority of circRNAs were exonic circRNAs in all samples, whereas the sense overlapping circRNAs just took up a small proportion. Differently, among differently expressed circRNAs (DE-circRNAs, fold change > 2, *p*-value < 0.05), the intergenic circRNAs took up the least (Fig. 3a). The details of the novel and known circRNAs in 5 isoforms were shown in Fig. 3b. The proportion rate among 5 isoforms of DE-circRNAs was further shown in Supplementary Fig. 1A. Among the exonic circRNAs, the mean length of known circRNAs were majorly concentrated on 200–300 nt (254 nt, Fig. 3c). Known circRNAs were distributed across all chromosomes, while DE-circRNAs were distributed across all chromosomes but chromosome 22 and chromosome Y. Significantly, the circRNAs across chromosome 1 and 2 were more enriched than other chromosomes (Fig. 3d). The dysregulated distribution of DE-circRNAs was also exhibited (Suppementary Fig. 1). Furthermore, the details of expression profiles of all circRNAs quantitated in our study were presented in Supplementary Data 1 and 2. The data of chromosomal location of the DE-circRNAs were shown in Supplementary Data 3.

### Annotation, visualization and functional clustering of DE-circRNAs

**Fig. 4:**
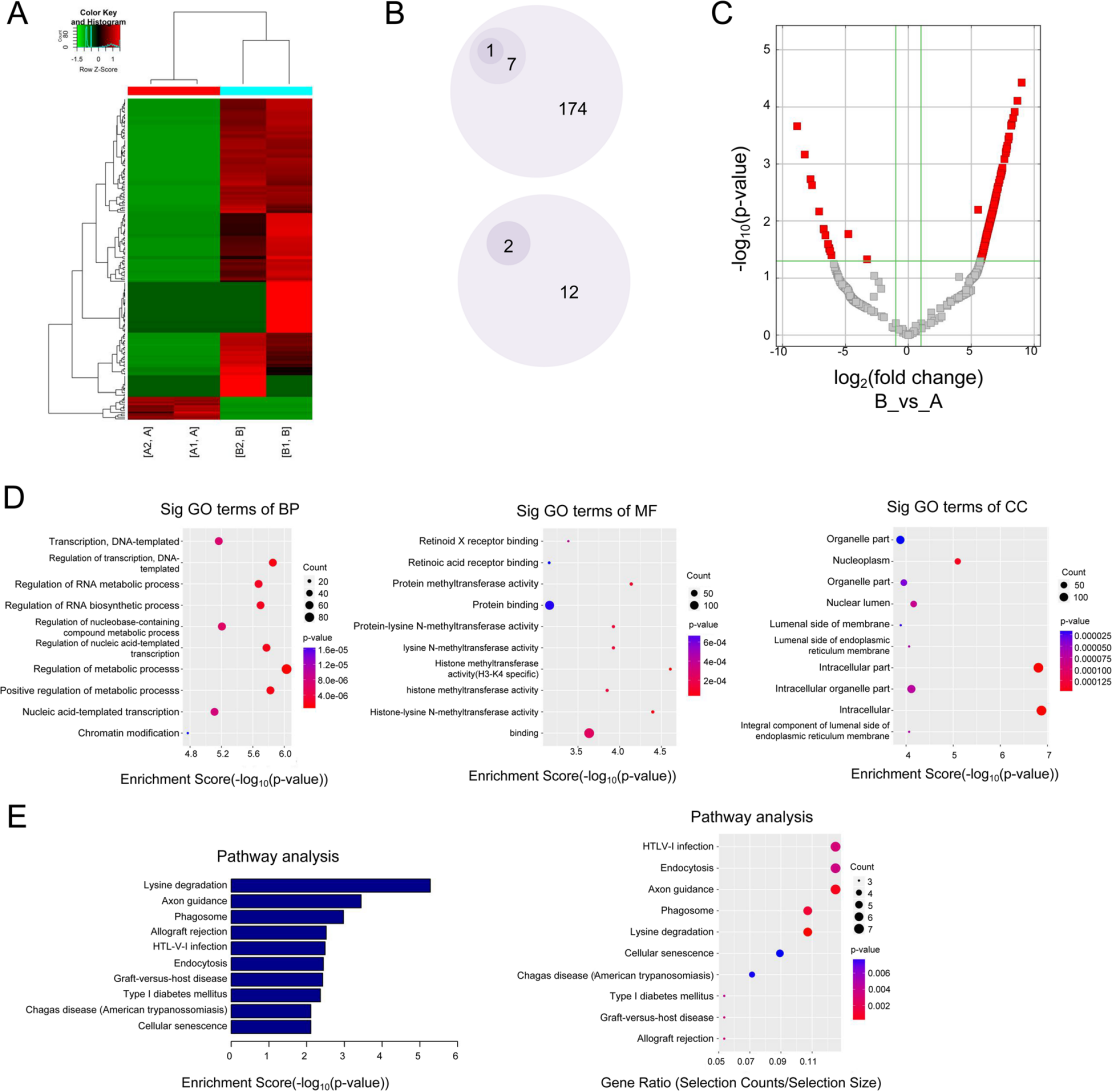
Annotation, visualization and functional clustering of DE-circRNAs. a Heatmap of the DE-circRNAs between group A and group B. **b** Bullet graph of the DE-circRNAs between two groups. **c** Volcano plot of the DE-circRNAs. The vertical lines correspond to fold-change, and the horizontal lines to *p*-value. Red rectangles represent DE-circRNAs (|fold change| > 2.0 and *p* < 0.05). Top 10 items of GO and KEGG enrichment of DE-circRNAs were represented in **d**, **e**

CircRNA expression levels changed in these exosomes. 196 of DE-circRNAs were identified and shown in heatmap, consisting of 182 up-regulated and 14 down-regulated circRNAs (Fig. 4a, fold change > 2, *p*-value < 0.05). Especially, all the down-regulated circRNAs were located on the chromosome M. We noted that 174 circRNAs were 6 folds significantly up-regulated, 7 circRNAs were 8 folds significantly up-regulated, and 1 circRNA (hsa_circ_0000284) were more than 8 folds significantly up-regulated. Moreover, 12 circRNAs were 6 folds significantly down-regulated, and 2 novel circRNAs (chrM:14131–15754- and chrM: 14131–115754+) were 8 folds significantly down-regulated (Fig. 4b). The volcano plot also revealed the dysregulated circRNAs clustered in red squares (Fig. 4c).

Five hundred eighty-six GO terms were enriched by analyzing the host genes of the up-regulated circRNAs, which contained biological process (BP), cellular component (CC) and molecular function (MF), and the top 10 terms were shown (Fig. 4d). It was revealed that genes relating to the regulation of metabolic process, binding and intracellular were most enriched in GO analysis. Twenty six enriched KEGG pathways were associated with lysine degradation, phagosome, axon guidance, allograft rejection, HTLV-I infection, endocytosis, Type I diabetes mellitus and Graft-versus-host disease (Fig. 4e). Furthermore, the details of all terms of GO and KEGG were shown in Supplementary Data 4 and 5.

### Experimental validation of circRNA-sequencing

**Fig. 5:**
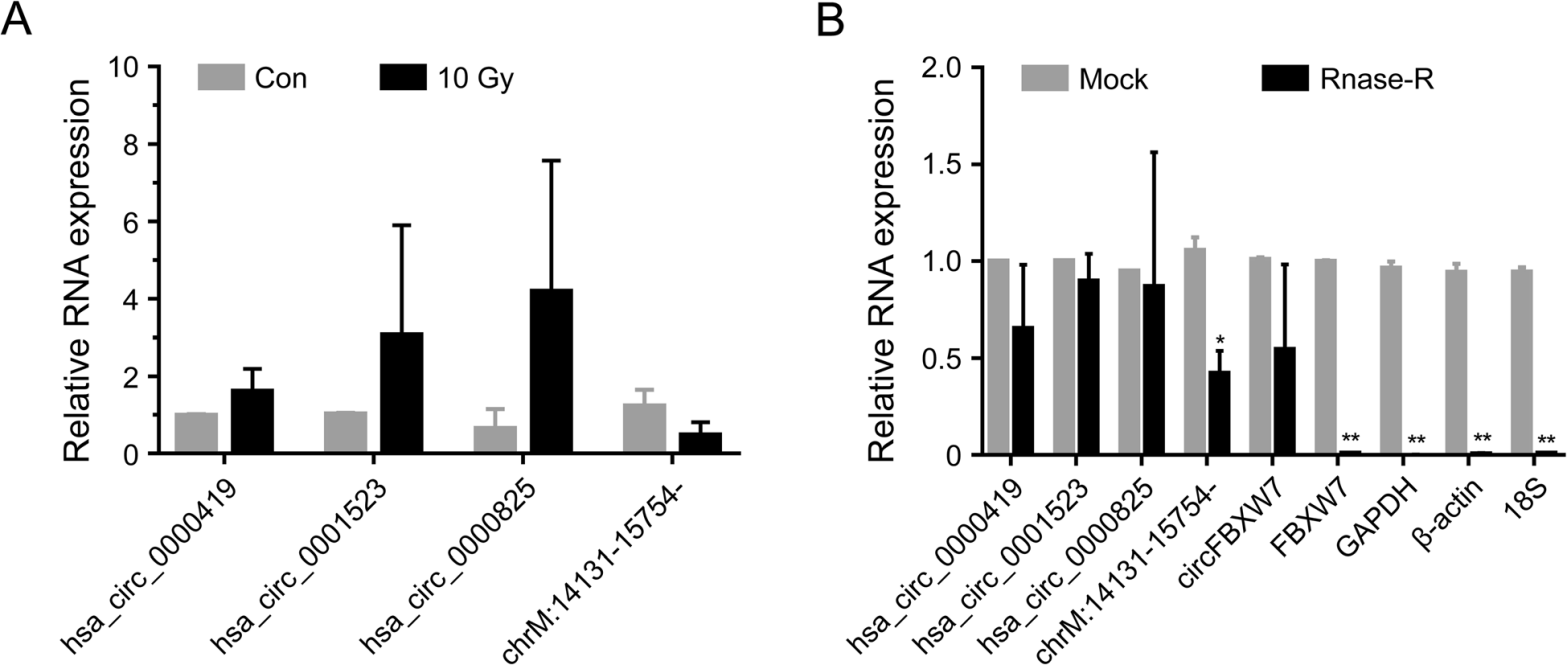
Validation of the circRNA-sequencing results. a Expression levels of hsa_ circ_0000419, hsa_circ_0001523, hsa_circ_0000825, and chrM: 14131–15,754- in the irradiation group (group B) with that in the non-irradiation group (group A). The results of quantitative real-time PCR (qRT-PCR) were evaluated by 2^-△△CT^ method. Results were represented as means ± standard deviation (SD). *p-value < 0.05. **b** CircRNAs were resistant to RNase R digestion, whereas the linear RNAs were sensitive to RNase R digestion. Four circRNAs were examined, which were qualified in expression level. And FBXW7, GAPDH, β-actin, and 18S rRNA were used as negative controls and known circRNA FBXW7 was used as a positive control. The results of quantitative Real-time PCR (qRT-PCR) were evaluated by 2^-△CT^ method. Results were represented as means ± standard deviation (SD) *p-value < 0.05, ** *p*-value < 0.001

Four circRNAs (hsa_circ_0000419, hsa_circ_0001523, hsa_circ_0000825, and chrM:14131–15754-) identified as DE-circRNAs by high-throughput sequencing were randomly selected for validation by qRT-PCR. The results were consistent with the sequencing results (Fig. 5a). We also selected the above mentioned circRNA, circFBXW7, and paired linear RNA FBXW7 [23] mRNA of common housekeeping genes (β-actin, GAPDH, and 18S rRNA) as controls for RNase R resistance experiment. The results revealed that the expression of all linear RNAs (FBXW7, β-actin, GAPDH and 18S rRNA) decreased after 1 h of RNase R digestion (** *p*-value < 0.001). This indicated that circRNAs were resistant to RNase R digestion, whereas linear RNAs were sensitive to R treatment (Fig. 5b).

### Prediction of circRNA-miRNA interaction network

**Fig. 6:**
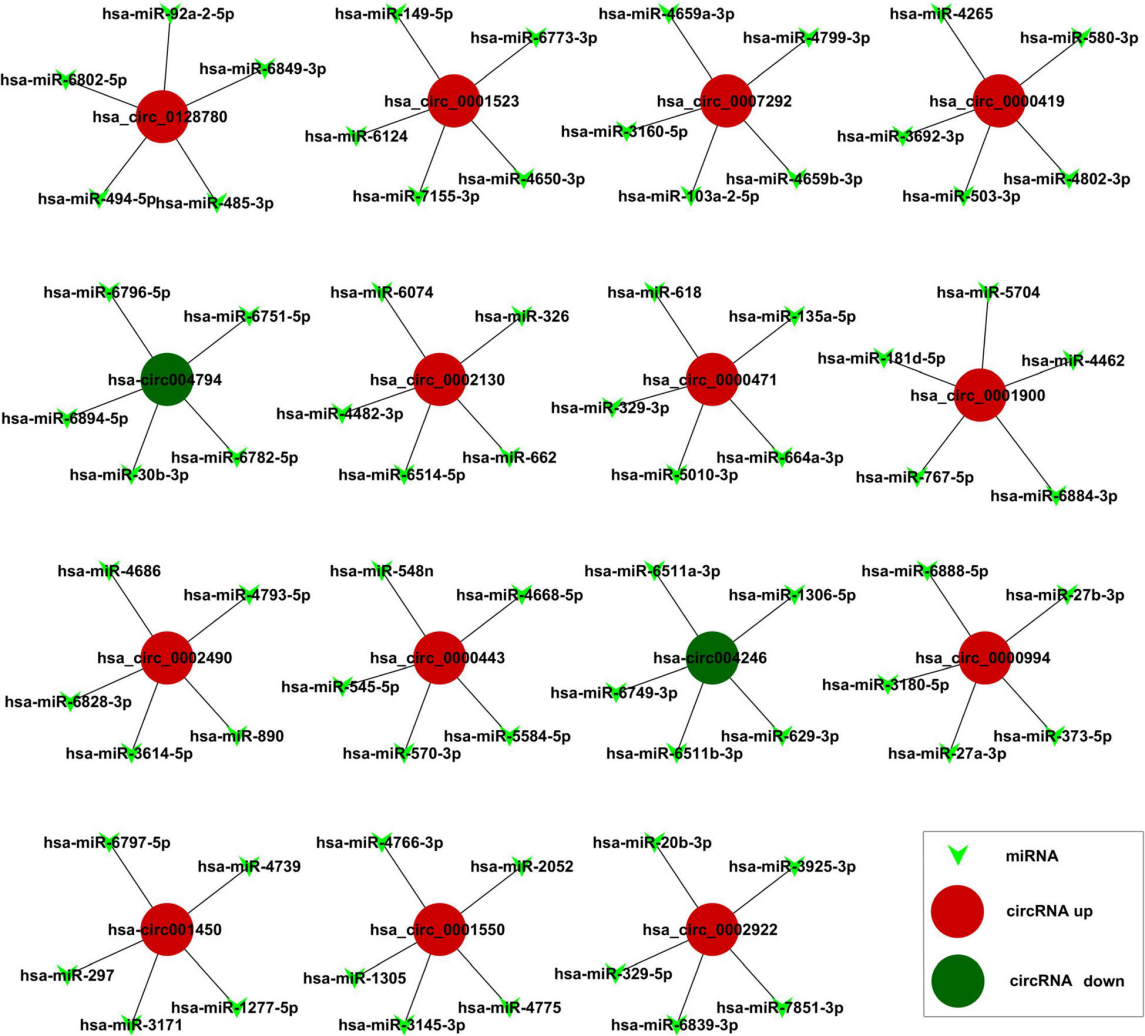
circRNA-miRNA network prediction. The top 15 significantly expressed circRNAs and top 5 predicted miRNAs were selected to generate a network map. The circRNA-miRNA network was built using bioinformatics online programs (TargetScan and miRBase). The red circle indicated the up-regulated circRNAs and the green ones indicated the down-regulated ones

CircRNAs could act as “miRNA sponge” to regulate miRNA level [17, 18]. To explore the function of circRNAs, the binding sites between DE-circRNAs and miRNAs were conducted by online databases. Thus, we selected the top 5 DE-circRNAs (*p*-value < 0.0001) to build a circRNA-miRNA interaction network, whose sequence might paired with the seed sequence of miRNA (Fig. 6). The circRNA-miRNA interaction analysis for all DE-circRNAs was shown in Supplementary Data 6.

### hsa_circ_0002130/hsa_miR_4482-3p/NBN interaction axis

**Fig. 7:**
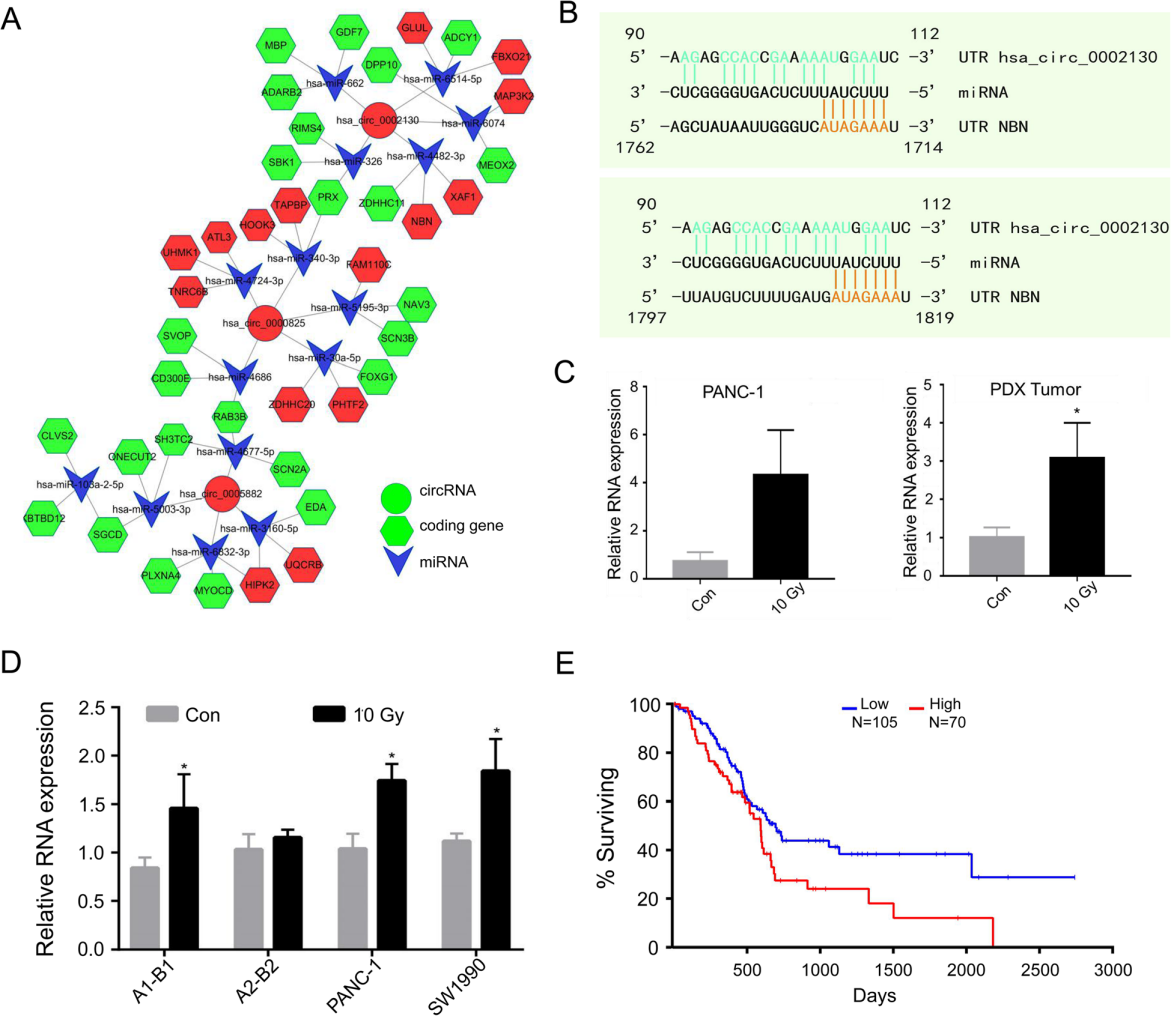
Hsa_circ_0002130-hsa_miR_4482-3p-NBN interaction axis. a The circRNA-miRNA-mRNA interaction network was predicted using online bioinformatics programs (Circinteractome, Circbank, TargetScan, and miRBase). The red circle indicated the up-regulated circRNAs, the green indicated the down-regulated ones, the arrow and the hexagon indicated miRNAs and target genes respectively. **b** The predicted binding sites between hsa_circ_0002130 and hsa_miR_4482-3p were exhibited. **c** The expression of hsa_circ_0002130 in PANC-1 cells (Left) and PDX tumors (Right) were examined respectively by qRT-PCR. **d** The expression of NBN was determined by qRT-PCR in samples of sequencing, PANC-1 and SW1990. *p-value < 0.05 and data indicated the mean ± SD. **e** The survival data from TCGA (Log rank *p*-value = 0.0234)

According to the highest binding force of miRNA to mRNA, 3 top known DE-circRNAs (hsa_circ_0002130, hsa_circ_0000825 and hsa_circ_0005882, *p*-value < 0.001) were chosen to construct the mRNA-miRNA-circRNA interaction network axis (Fig. 7a), and the newly circRNAs were shown in Supplementary Fig. 2. For example, we displayed the hsa_circ_0002130 to further observe the influence of target genes. In previous studies, the irradiation-induced cellular damage included DNA strand breaks [24]. Nibrin (NBN) is the gene whose product is associated with DNA double-strand break repair and DNA damage-induced check point activation [25]. Therefore, we supposed NBN could be related with the repopulation in pancreatic cancer. Here, NBN might be regulated by hsa_miR_4482-3p, a hsa_circ_0002130 binding miRNA, through two possible binding sites (Fig. 7b). To further validate the expression changes of the axis, we performed qPCR assay. The expression level of hsa_circ_0002130 in cancer cell-derived exosomes was upregulated after radiation (Fig. 7c (Left)). RNase R experiments were shown in the Supplementary Fig. 1C. To further validate the change of hsa_circ_0002130 in vivo, we performed qPCR assay in exosomes derived from plasma of the irradiated/unirradiated PDX tumor, which was generated in our previous work [26]. We identified that exosomal hsa_circ_0002130 was apparently higher in plasma from the irradiated mice compared with that in the untreated group (Fig. 7c (Right)). Furthermore, mRNA level of NBN was up-regulated in exosomes during irradiation (Fig. 7d). According to TCGA, the survival curve for NBN was shown in Fig. 7e (Log rank *p*-value = 0.0234), which showed the survival probability of patients with the higher level expression of NBN was shorter than the lower ones.

## Discussion

In our study, 12,572 circRNAs were identified, and 3580 circRNAs were annotated as the known circRNAs. We obtained the reads of circRNAs from 5 parts depending on the location of the chromosome, including exonic, intronic, sense overlapping, intergenic and antisense, which were similar to the publication [13]. Different circRNAs were produced by selecting different splicing sites and existed mainly in exons. However, we also found most of the identified circRNAs (71.52%) were novel, which mainly concentrated on four parts except the exon (Fig. 3b). Besides, the mean length of these identified exonic circRNAs was focus on 200–300 nt (Fig. 3c). Interestingly, it was reported that circRNAs releasing could be influenced by their own length, which indicated that the length of circRNAs might affect its formation [27]. Moreover, the unique structure of circRNA without a poly(A)-tail rendered themselves highly insensitive to ribonuclease. We showed that circRNAs were more stable than linear RNAs through RNase R digestion. Alternatively, Ribo-zero could also be used to circRNA validation [28].

The results in BP indicated that the highly expressed target genes might be involved in regulation of metabolic process, RNA biosynthetic and metabolic process, and nucleic acid-templated transcription. These GO terms might be associated with nucleotide metabolism, which was involved in radiotherapy [29]. Refer to MF, “Binding”, “protein methyltransferase activity”, “histone methyltransferase activity”, “histone methyltransferase activity (H3-K4 specific)” and “histone-lysine N-methyltransferase activity” were highly enriched. As reported, VDR and p53 in irradiated HEK 293 T was related to various biological effects, which mediated gene transcription [30]. Furthermore, aberrant methylation could lead to dysregulation of related transcription-factor genes, which might be closely bound up to irradiation [31, 32]. KEGG analysis indicated that the lysine degradation was important, which was consistent with the results of GO analysis. Lysine degradation mainly participated in the control of gene expression and transcription. These findings indicated the cargos in exosomes could regulate different pathway and biological processes concerning cell proliferation and irradiation. However, further experimental verification is still needed, especially onto tumor repopulation owing to radiotherapy.

Refer to miRNA sponge, numerous miRNA binding sites were predicted by Circinteractome, Circbank, Miranda and TargetScan, which revealed circRNAs could interact with miRNAs. Based on bioinformatics, mRNA-miRNA-circRNA regulatory network was constructed to clarify the interactions between molecules. As shown (Fig. 7a), the hsa_circ_0002130/hsa_miR_4482-3p/NBN interaction axis was involved in the regulatory network. NBN gene is a member of the MRE11/RAD50 double-strand break repair complex. Several studies confirmed that NBN was crucially involved in DNA double strand fracture repairing and DNA damage-induced checkpoint activation [33]. The expression level of hsa_circ_0002130 was also up-regulated during irradiation, consistent with the expression pattern of NBN. Given the potential binding sites among hsa_circ_0002130/hsa_miR_4482-3p/NBN, hsa_circ_0002130 was considered to promote the expression of NBN through the absorption of hsa-miR-4482-3p. As we recently found that irradiated dying tumor cells-derived exosomes promoted DNA damage repair and the survival of damaged tumor repopulating cells [26] the exosomal hsa_circ_0002130 might also mediate the vital role of the exosomes. Thus, further works could be directed to identify the role of dying tumor cell-derived exosomal hsa_circ_0002130 in potentiating tumor repopulation via promoting DNA damage repair, which was deduced to be mediated by the hsa_circ_0002130/hsa_miR_4482-3p/NBN axis. Remarkably, some circRNAs were also shown to be translated into proteins [34, 35]. Thus, the underlying mechanisms still needed further investigations.

Nevertheless, there are still several drawbacks inherent to our study. The small sample size was for RNA-sequencing, and samples from the patients were hard for us to collect. Therefore, the influence from the patients should be further validated. On the other hand, even if we believe that they worked, we did not perform further experiments to confirm the regulatory relationship among circRNAs, miRNAs and mRNAs, as well as other functions of circRNAs.

## Conclusions

Hereinabove, we first identified and annotated 12,572 circRNAs in exosomes of human pancreatic cancer cells upon irradiation using RNA-seq analysis. Among them, the DE-circRNAs were associated with methylation, which was in accord with the signaling pathways. In further prediction, hsa_circ_0002130 could target NBN by combing with the hsa_miR_4482-3p and high expression of NBN revealed a worse survival rate.

## Supplementary information

**Additional file 1: Supplementary Data 1.** Detail information of all the circRNAs identified in this study see Full Excel version.

**Additional file 2: Supplementary Data 2.** Detail information of the differentially expressed circRNAs identified in this study – see Full Excel version.

**Additional file 3: Supplementary Data 3.** The sequence of DE-circRNAs in this study - see Full text version.

**Additional file 4: Supplementary Data 4.** Details of the GO enrichment with statistical significance – see Full Excel version.

**Additional file 5: Supplementary Data 5.** Details of the KEGG enrichment with statistical significance – see Full Excel version.

**Additional file 6: Supplementary Data 6.** The mRNA-miRNA-circRNA interaction analysis for all DE-circRNAs – see Full Excel version.

**Additional file 7: Supplementary Table 1.** Quality control (QC) of RNA experiment. **Supplementary Table 2.** Quality assessment of sequencing library. **Supplementary Table 3.** Analysis of sequencing reads. **Supplementary Table 4.** Primer sequences used in this study.

**Additional file 8: Supplementary Figure 1.** Different circRNAs in samples. (A) The further proportionate among 5 isoforms of DE-circRNAs. (B) The further dysregulated chromosomal distribution of DE-circRNAs. (C) RNase experiment samples were examined by qRT-PCR.

**Additional file 9: Supplementary Figure 2.** The predicted mRNA-miRNA-circRNA interaction network. The interaction network of mRNA-miRNA-circRNA was predicted using bioinformatics online programs (CircInteractome, circBank, TargetScan, and miRBase). The red circle indicated up-regulated circRNA, the green indicated down-regulated, the arrow and the hexagon indicated miRNA and target gene respectively.

## Data Availability

The RNA-Seq data generated during the current study are available in the GEO database (GSE132678) (https://www.ncbi.nlm.nih.gov/geo/query/acc.cgi?acc=GSE132678). All other relevant data is available within the manuscript or its accompanying supplementary material.

## Abbreviations

circRNAs: Circular RNAs
NGS: Next generation sequencing
qRT-PCR: Quantitative real-time reverse transcription PCR
miRNA: MicroRNA
lncRNA: Long non-coding RNA
MREs: miRNA recognition elements
GO: Gene ontology
KEGG: Kyotoencyclopedia of genes and genomes
DE-circRNAs: Differentially expressed circRNAs
TEM: Transmission electron microscope
BP: Biological process
CC: Cellular component
MF: Molecular function

## References

[1] Bray F, Ferlay J, Soerjomataram I, Siegel RL, Torre LA, Jemal A. Global cancer statistics 2018: GLOBOCAN estimates of incidence and mortality worldwide for 36 cancers in 185 countries. CA Cancer J Clin. 2018. 10.3322/caac.21492. 10.3322/caac.21492 30207593

[2] Palta M, Godfrey D, Goodman KA, Hoffe S, Dawson LA, Dessert D, Hall WA, Herman JM, Khorana AA, Merchant N, Parekh A, Patton C, Pepek JM, Salama JK, Tuli R, Koong AC. Radiation therapy for pancreatic Cancer: executive summary of an ASTRO clinical practice guideline. Pract Radiat Oncol. 2019. 10.1016/j.prro.2019.06.016. 10.1016/j.prro.2019.06.016 31474330

[3] Venkatesulu BP, Hsieh CE, Sanders KL, Krishnan S. Recent advances in radiation therapy of pancreatic cancer. F1000Res. 2018. 10.12688/f1000research.16272.1. 10.12688/f1000research.16272.1 PMC6305239 30613390

[4] Jiang MJ, Gu DN, Dai JJ, Huang Q ,Tian, L. Dark Side of Cytotoxic Therapy: Chemoradiation-Induced Cell Death and Tumor Repopulation. Trends Cancer. 2020. 10.1016/j.trecan.2020.01.018. 10.1016/j.trecan.2020.01.018 32348737

[5] Kim JJ, Tannock IF. Repopulation of cancer cells during therapy: an important cause of treatment failure. Nat Rev Cancer. 2005. 10.1038/nrc1650. 10.1038/nrc1650 15965493

[6] Fang C, Dai CY, Mei Z, Jiang MJ, Gu DN, Huang Q, Tian L. MicroRNA-193a stimulates pancreatic cancer cell repopulation and metastasis through modulating TGF-β2/TGF-βRIII signalings. J Exp Clin Cancer Res. 2018. 10.1186/s13046-018-0697-3. 10.1186/s13046-018-0697-3 PMC5809917 29433538

[7] Huang Q, Li F, Liu X, Li W, Shi W, Liu FF, O'Sullivan B, He Z, Peng Y, Tan AC, Zhou L, Shen J, Han G, Wang XJ, Thorburn J, Thorburn A, Jimeno A, Raben D, Bedford JS, Li CY. Caspase 3-mediated stimulation of tumor cell repopulation during cancer radiotherapy. Nat Med. 2011. 10.1038/nm.2385. 10.1038/nm.2385 PMC3132290 21725296

[8] Ma J, Cheng J, Gong Y, Tian L, Huang Q. Downregulation of Wnt signaling by sonic hedgehog activation promotes repopulation of human tumor cell lines. Dis Model Mech. 2015. 10.1242/dmm.018887. 10.1242/dmm.018887 PMC4381337 25713298

[9] van Niel G, D'Angelo G, Raposo G. Shedding light on the cell biology of extracellular vesicles. Nat Rev Mol Cell Biol. 2018. 10.1038/nrm.2017.125. 10.1038/nrm.2017.125 29339798

[10] Pavlyukov MS, Yu H, Bastola S, Minata M, Shender VO, Lee Y, Zhang S, Wang J, Komarova S, Wang J, Yamaguchi S, Alsheikh HA, Shi J, Chen D, Mohyeldin A, Kim SH, Shin YJ, Anufrieva K, Evtushenko EG, Antipova NV, Arapidi GP, Govorun V, Pestov NB, Shakhparonov MI, Lee LJ, Nam DH, Nakano I. Apoptotic cell-derived extracellular vesicles promote malignancy of glioblastoma via intercellular transfer of splicing factors. Cancer Cell. 2018. 10.1016/j.ccell.2018.05.012. 10.1016/j.ccell.2018.05.012 PMC6048596 29937354

[11] Becker A, Thakur BK, Weiss JM, Kim HS, Peinado H, Lyden D. Extracellular vesicles in cancer: cell-to-cell mediators of metastasis. Cancer Cell. 2016. 10.1016/j.ccell.2016.10.009. 10.1016/j.ccell.2016.10.009 PMC5157696 27960084

[12] Vicens Q, Westhof E. Biogenesis of circular RNAs. Cell. 2014. 10.1016/j.cell.2014.09.005. 10.1016/j.cell.2014.09.005 25259915

[13] Memczak S, Jens M, Elefsinioti A, Torti F, Krueger J, Rybak A, Maier L, Mackowiak SD, Gregersen LH, Munschauer M, Loewer A, Ziebold U, Landthaler M, Kocks C, le Noble F, Rajewsky N. Circular RNAs are a large class of animal RNAs with regulatory potency. Nature. 2013. 10.1038/nature11928. 10.1038/nature11928 23446348

[14] Zheng Q, Bao C, Guo W, Li S, Chen J, Chen B, Luo Y, Lyu D, Li Y, Shi G, Liang L, Gu J, He X, Huang S. Circular RNA profiling reveals an abundant circHIPK3 that regulates cell growth by sponging multiple miRNAs. Nat Commun. 2016. 10.1038/ncomms11215. 10.1038/ncomms11215 PMC4823868 27050392

[15] Hansen TB, Jensen T, Clausen BH, Bramsen JB, Finsen B, Damgaard CK, Kjems J. Natural RNA circles function as efficient microRNA sponges. Nature. 2013. 10.1038/nature11993. 10.1038/nature11993 23446346

[16] Bezzi M, Guarnerio J, Pandolfi PP. A circular twist on microRNA regulation. Cell Res. 2017. 10.1038/cr.2017.136. 10.1038/cr.2017.136 PMC5717405 29086764

[17] Zhang H, Zhu L, Bai M, Liu Y, Zhan Y, Deng T, Yang H, Sun W, Wang X, Zhu K, Fan Q, Li J, Ying G, Ba Y. Exosomal circRNA derived from gastric tumor promotes white adipose browning by targeting the miR-133/PRDM16 pathway. Int J Cancer. 2019. 10.1002/ijc.31977. 10.1002/ijc.31977 30412280

[18] Li Z, Yanfang W, Li J, Jiang P, Peng T, Chen K, Zhao X, Zhang Y, Zhen P, Zhu J, Li X. Tumor-released exosomal circular RNA PDE8A promotes invasive growth via the miR-338/MACC1/MET pathway in pancreatic cancer. Cancer Lett 2018. 10.1016/j.canlet.2018.04.035. 10.1016/j.canlet.2018.04.035 29709702

[19] Bailey P, Chang DK, Nones K, Johns AL, Patch AM, Gingras MC, Miller DK, Christ AN, Bruxner TJ, Quinn MC, Nourse C, Murtaugh LC, Harliwong I, Idrisoglu S, Manning S, Nourbakhsh E, Wani S, Fink L, Holmes O, Chin V, Anderson MJ, Kazakoff S, Leonard C, Newell F, Waddell N, Wood S, Xu Q, Wilson PJ, Cloonan N, Kassahn KS, Taylor D, Quek K, Robertson A, Pantano L, Mincarelli L, Sanchez LN, Evers L, Wu J, Pinese M, Cowley MJ, Jones MD, Colvin EK, Nagrial AM, Humphrey ES, Chantrill LA, Mawson A, Humphris J, Chou A, Pajic M, Scarlett CJ, Pinho AV, Giry-Laterriere M, Rooman I, Samra JS, Kench JG, Lovell JA, Merrett ND, Toon CW, Epari K, Nguyen NQ, Barbour A, Zeps N, Moran-Jones K, Jamieson NB, Graham JS, Duthie F, Oien K, Hair J, Grützmann R, Maitra A, Iacobuzio-Donahue CA, Wolfgang CL, Morgan RA, Lawlor RT, Corbo V, Bassi C, Rusev B, Capelli P, Salvia R, Tortora G, Mukhopadhyay D, Petersen GM, Australian Pancreatic Cancer Genome Initiative, Munzy DM, Fisher WE, Karim SA, Eshleman JR, Hruban RH, Pilarsky C, Morton JP, Sansom OJ, Scarpa A, Musgrove EA, Bailey UM, Hofmann O, Sutherland RL, Wheeler DA, Gill AJ, Gibbs RA, Pearson JV, Waddell N, Biankin AV, Grimmond SM. Genomic analyses identify molecular subtypes of pancreatic cancer. Nature. 2016. 10.1038/nature16965.

[20] Shen GQ, Aleassa EM, Walsh RM, Morris-Stiff G. Next-generation sequencing in pancreatic Cancer. Pancreas. 2019. 10.1097/MPA.0000000000001324. 10.1097/MPA.0000000000001324 31206465

[21] Shelke GV, Lässer C, Gho YS, Lötvall J. Importance of exosome depletion protocols to eliminate functional and RNA-containing extracellular vesicles from fetal bovine serum. J Extracell Vesicles. 2014. 10.3402/jev.v3.24783. 10.3402/jev.v3.24783 PMC4185091 25317276

[22] Greening DW, Xu R, Ji H, Tauro BJ, Simpson RJ. A protocol for exosome isolation and characterization: evaluation of ultracentrifugation, density-gradient separation, and immunoaffinity capture methods. Methods Mol Biol. 2015. 10.1007/978-1-4939-2550-6_15. 10.1007/978-1-4939-2550-6_15 25820723

[23] Yang Y, Gao X, Zhang M, Yan S, Sun C, Xiao F, Huang N, Yang X, Zhao K, Zhou H, Huang S, Xie B, Zhang N. Novel role of FBXW7 circular RNA in repressing glioma tumorigenesis. J Natl Cancer Inst. 2018. 10.1093/jnci/djx166. 10.1093/jnci/djx166 PMC6019044 28903484

[24] Baskar R, Lee KA, Yeo R, Yeoh KW. Cancer and radiation therapy: current advances and future directions. Int J Med Sci. 2012. 10.7150/ijms.3635. 10.7150/ijms.3635 PMC3298009 22408567

[25] Pennisi R, Antoccia A, Leone S, Ascenzi P, di Masi A. Hsp90α regulates ATM and NBN functions in sensing and repair of DNA double-strand breaks. FEBS J. 2017. 10.1111/febs.14145. 10.1111/febs.14145 28631426

[26] Jiang MJ, Chen YY, Dai JJ, Gu DN, Mei Z, Liu FR, Huang Q, Tian L. Dying tumor cell-derived exosomal miR-194-5p potentiates survival and repopulation of tumor repopulating cells upon radiotherapy in pancreatic cancer. Mol Cancer. 2020. 10.1186/s12943-020-01178-6. 10.1186/s12943-020-01178-6 PMC7104536 32228703

[27] Huang C, Liang D, Tatomer DC, Wilusz JE. A length-dependent evolutionarily conserved pathway controls nuclear export of circular RNAs. Genes Dev. 2018. 10.1101/gad.314856.118. 10.1101/gad.314856.118 PMC6004072 29773557

[28] Vo JN, Cieslik M, Zhang Y, Shukla S, Xiao L, Zhang Y, Wu YM, Dhanasekaran SM, Engelke CG, Cao X, Robinson DR, Nesvizhskii AI, Chinnaiyan AM. The landscape of circular RNA in cancer. Cell. 2019. 10.1016/j.cell.2018.12.021. 10.1016/j.cell.2018.12.021 PMC6601354 30735636

[29] Gunda V, Souchek J, Abrego J, Shukla SK, Goode GD, Vernucci E, Dasgupta A, Chaika NV, King RJ, Li S, Wang S, Yu F, Bessho T, Lin C, Singh PK. MUC1-mediated metabolic alterations regulate response to radiotherapy in pancreatic cancer. Clin Cancer Res. 2017. 10.1158/1078-0432.CCR-17-1151. 10.1158/1078-0432.CCR-17-1151 PMC5626603 28720669

[30] Pemsel A, Rumpf S, Roemer K, Heyne K, Vogt T, Reichrath J. Tandem affinity purification and nano HPLC-ESI-MS/MS reveal binding of vitamin D receptor to p53 and other new interaction partners in HEK 293T cells. Anticancer Res. 2018. 10.21873/anticanres.12341. 10.21873/anticanres.12341 29374759

[31] Daino K, Nishimura M, Imaoka T, Takabatake M, Morioka T, Nishimura Y, Shimada Y, Kakinuma S. Epigenetic dysregulation of key developmental genes in radiation-induced rat mammary carcinomas. Int J Cancer. 2018. 10.1002/ijc.31309. 10.1002/ijc.31309 29435983

[32] Kejík Z, Jakubek M, Kaplánek R, Králová J, Mikula I, Martásek P, Král V. Epigenetic agents in combined anticancer therapy. Future Med Chem. 2018. 10.4155/fmc-2017-0203. 10.4155/fmc-2017-0203 29676175

[33] Gatei M, Young D, Cerosaletti KM, Desai-Mehta A, Spring K, Kozlov S, Lavin MF, Gatti RA, Concannon P, Khanna K. ATM-dependent phosphorylation of nibrin in response to radiation exposure. Nat Genet. 2000. 10.1038/75508. 10.1038/75508 10802669

[34] Yang Y, Fan X, Mao M, Song X, Wu P, Zhang Y, Jin Y, Yang Y, Chen LL, Wang Y, Wong CC, Xiao X, Wang Z. Extensive translation of circular RNAs driven by N^6^-methyladenosine. Cell Res. 10.1038/cr.2017.31. 10.1038/cr.2017.31 PMC5520850 28281539

[35] Legnini I, Di Timoteo G, Rossi F, Morlando M, Briganti F, Sthandier O, Fatica A, Santini T, Andronache A, Wade M, Laneve P, Rajewsky N, Bozzoni I. Circ-ZNF609 is a circular RNA that can be translated and functions in myogenesis. Mol Cell. 2017. 10.1016/j.molcel.2017.02.017. 10.1016/j.molcel.2017.02.017 PMC5387670 28344082

