# Supplementary material for: Analysis of exosomal circRNAs upon irradiation in pancreatic cancer cell repopulation
Source: BMC Med Genomics. 2020 Jul 29;13:107. doi: 10.1186/s12920-020-00756-3 (PMC7391519; doi:10.1186/s12920-020-00756-3)
Supplement: Supplementary file 7 — Additional file 7: Supplementary Table 1. Quality control (QC) of RNA experiment. Supplementary Table 2. Quality assessment of sequencing library. Supplementary Table 3. Analysis of sequencing reads. Supplementary Table 4. Primer sequences used in this study. [file 12920_2020_756_MOESM7_ESM.docx]

**Supplementary Table 1. Quality Control (QC) of RNA Experiment**

| **Sample** | **OD260/280** | **Conc.(ng/μl)** | **Volume(μl)** | **Quantity(μg)** |
| --- | --- | --- | --- | --- |
| A1 | 1.59 | 152.03 | 13 | 1.98 |
| A2 | 1.66 | 100.28 | 13 | 1.30 |
| B1 | 1.54 | 130.01 | 13 | 1.69 |
| B2 | 1.64 | 142.42 | 13 | 1.85 |

**Supplementary Table 2.** **Quality Assessment of Sequencing Library**

| **Sample** | **Size(bp)** | **Conc.(ng/μl)** | **Conc.(nmol/L)** | **Volume(μl)** | **Total Amount(ng)** |
| --- | --- | --- | --- | --- | --- |
| A1 | 299 | 5.09 | 25.8 | 10 | 50.9 |
| A2 | 310 | 6.02 | 29.4 | 10 | 60.2 |
| B1 | 306 | 5.81 | 28.8 | 10 | 58.1 |
| B2 | 275 | 5.61 | 30.9 | 10 | 56.1 |

**Supplementary Table 3. Analysis of Sequencing Reads**

| **Sample** | **Raw Reads** | **Q30** | **Clean Reads** | **Clean Ratio** | **Mapped Reads** | **Mapped Ratio** | **CircRNA Number** |
| --- | --- | --- | --- | --- | --- | --- | --- |
| A1 | 83,597,758 | 93.75% | 83,514,968 | 99.90% | 74,611,190 | 89.34% | 2052 |
| A2 | 100,327,034 | 94.18% | 100,202,554 | 99.88% | 89,675,186 | 89.49% | 2485 |
| B1 | 95,908,420 | 94.40% | 95,781,818 | 99.87% | 87,512,128 | 91.37% | 3493 |
| B2 | 102,744,612 | 93.60% | 102,457,788 | 99.72% | 88,841,466 | 86.71% | 2066 |

**Supplementary Table 4.** **Primer sequences used in this study**

| **Transcript ID** | **Primer Sequence** |
| --- | --- |
| **Up-regulated circRNAs:** |  |
| hsa_circ_0000419 | F: 5’- GCTGTTCTGGAGGCTGTGGA-3’  R: 5’- CGTCCTGTCCATTGTGGGCT-3’ |
| hsa_circ_0001523 | F: 5’- TCTTGGGCCCTGTGAACCTG-3’  R: 5’- GGCGCTGGCACTGTAAACAG-3’ |
| hsa_circ_0000825 | F: 5’- ACAGGCCCTCCAGAATGAGC-3’  R: 5’- TTCTCGCCGAAGTTCCTGCA-3’ |
| chrM:14131-15754+ | F: 5’- GCCGCAGACCTCCTCATTCT-3’  R: 5’-AGGAGAGGGGTCAGGGTTGA-3’ |
| **Down-regulated circRNAs:** |  |
| chrM:14131-15754- | F: 5’-AGGAGAGGGGTCAGGGTTGA-3’  R: 5’- GCCGCAGACCTCCTCATTCT-3’ |
| **Common-expressed circRNAs:** |  |
| circFBXW7 | F: 5’- CAGTGTCACGAACTCCAGTA-3’  R: 5’- CACATTCCTCTGACCCAGTA-3’ |
| Linear RNAs: |  |
| FBXW7 | F: 5’-TTACAAAAACAAAATCCGGAGTCT-3’  R: 5’-TTTTCCTCTTCCTGGGTCTT-3’ |
| **Housekeeping gene:** |  |
| GAPDH | F: 5’-CACTAGGCGCTCACTGTTCTC-3’  R: 5’-GACTCCACGACGTACTCAGC-3’ |
| β-actin | F: 5’-ACAGAGCCTCGCCTTTGCCGAT-3’  R: 5’- CTTGCACATGCCGGAGCCGTT-3’ |
| 18S | F: 5’- GGCCCTGTAATTGGAATGATGC-3’  R: 5’- CCAAGATCCAACTACGAGCTT-3’ |
