# Supplementary figures and images for: Analysis of exosomal circRNAs upon irradiation in pancreatic cancer cell repopulation
Source: BMC Med Genomics. 2020 Jul 29;13:107. doi: 10.1186/s12920-020-00756-3 (PMC7391519; doi:10.1186/s12920-020-00756-3)

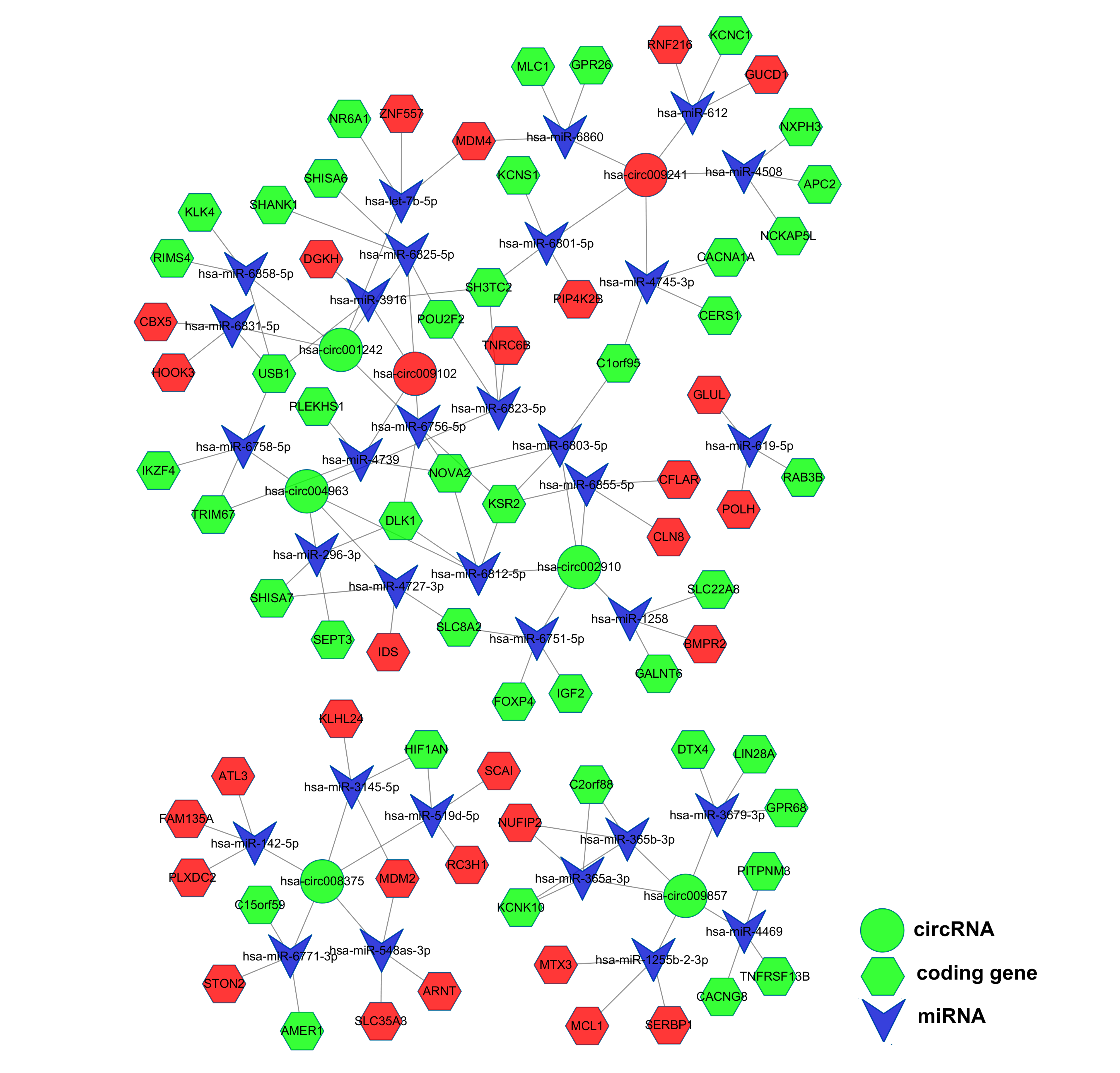

Supplement: Supplementary file 9 — Additional file 9: Supplementary Figure 2. The predicted mRNA-miRNA-circRNA interaction network. The interaction network of mRNA-miRNA-circRNA was predicted using bioinformatics online programs (CircInteractome, circBank, TargetScan, and miRBase). The red circle indicated up-regulated circRNA, the green indicated down-regulated, the arrow and the hexagon indicated miRNA and target gene respectively. [file 12920_2020_756_MOESM9_ESM.tif]
